# Supplementary material for: Learning curve and surgical outcome of robotic assisted colorectal surgery with ERAS program
Source: Sci Rep. 2022 Nov 29;12:20566. doi: 10.1038/s41598-022-24665-w (PMC9709162; doi:10.1038/s41598-022-24665-w)
Supplement: Supplementary file 1 — Supplementary Information 1. [file 41598_2022_24665_MOESM1_ESM.zip › LSC Raw data-Ñ[▒K/109/H_ñw╝╨░O▒Kñσ.pdf]

正式摘要(電子簽章：000000206180392)		
W52 / W52 ☐英文標題(注意:無英文姓名-請補病人基本檔!)	☐【讀取】憑證	

臺中榮民總醫院
出院病歷摘要

轉入號 : 轉出號 :

[redacted]

**(8)**流水編號	
**(9)**入院日期	109/12/23 (2020) 直腸外科 W52 - 171
**(10)**轉科日期	
**(11)**出院日期	109/12/28 (2020) 住院天數計 5 日

診斷

- 入院 :Sigmoid colon cancer
- 出院 :Sigmoid colon cancer s/p laparoscopic anterior resection on 2020/12/24

主訴

- positive stool occult blood was found in health examination

病史

- This is a 63 y/o man didn't have special past history. Positive stool OB was found in health examination. He then went to our CRS OPD where colonoscope revealed a huge polyp FAV 25cm and biopsy report showed malignancy. Thus, he was admitted for surgical treatment.

體檢發現

- Anal inspection:
  Recto digital examination:
  Sigmoidoscopy:
  Anoscopy:
  Abdomen:
  - General appearance :
    - easy looking , conscious clear , without cardiopulmonary distress
    - Vital signs自動帶入date 體溫(BT) 脈搏(P) 呼吸(R) 血壓(BP) 身高(BH) 體重(BW) BMI
      20201223 36.4℃ 75次/分 18次/分 160/59.4mmHg 160cm 59.4kg 23.2
  - HEENT :
    - Eyes: no anemic conjunctiva, no icteric sclera, normal EOM
    - ENT: no otorrhea, no rhinorrhea, no erythematous mucosa, no injected tonsil, no deformity
  - Neck: supple, freely movable, trachea midline,no deformity, no engorged jugular vein , no palpable thyroid or lymph node enlargement
  - Chest:
    - Inspection: Grossly normal, symmetric and fully expansion, no accessory muscle used, no operation scar, no spider angiomata, no gynecomastia
    - Palpation: no palpable mass or nodule, no subcutaneous emphysema, no tenderness
    - Ausculation: clear breathing sound
  - Heart:
    - Percussion: normal heart size
    - Ausculation: regular heart beat, normal S1, S2, no gallop, no murmur
  - Abdomen:
    - Inspection: in
      - flat contour, symetrically, no scar, no stria, no dilated veins, no rashes
    - Ausculation: normoactive bowel sound, no abdominal bruits

-
  - palpation: soft, no muscle guarding or rigidity, no local tenderness, no rebounding pain, no palpable mass, no palpable pulsation
  - Percussion: no shifting dulllness, no hepatomegaly, no splenomagaly
-

Peripheral pulsation :	CA	BA	RA	FA	PA	PTA	DPA
Right side :	++	++	++	++	++	++	++/+++
Left side :	++	++	++	++	++	++	++/+++

- Genito-anus: no tenderness or palpable mass lesion or rectal shelf, normal external genitalia, normal anal tone, brown stool

CRS CONDITION

-
  - Lung

手術日期及方法

- 手術日期及方法20201224 Laparoscopic Anterior resection and anastomosis - malignant

住院治療經過

- After admission, operation with laparoscopic anterior resection was done on 2020/12/24. He started feeding under ERAS protocol. Under the stable condition, he was discharged on 12/28.

合併症

- nil

一般檢查

- 累積報告 :

DATE	TIME	ALB	TP	BIL.T	BIL.D	ALKP	AST	ALT	LDH	CHOL	TG	HDL-C	LDL	UA	NH3	AMY
1091223	1451	4.5		0.84	0.18		35	23								
1091224	1331	3.6		0.9	0.2	79	31	19								

DATE	TIME	WBC	RBC	HGB	HCT	MCV	PLT	BLST	NEUT	BAND	SEG	LYM	MON	EOS	BAS
1091223	1455	9520	4.67	14.7	42.1	90.1	170		77.7			17.5	4.0	0.3	0.5
1091224	1334	14820		12.6	36.5		151		91.7			3.8	4.3	0.0	0.2

DATE	TIME	NA	K	CL	CA	BUN	CREAT	Mg	P	CRP	ESR	PCT	LIPASE	LACTATE
1091223	1451	140	4.6	103	9.3	18	1.32							
1091224	1331	140	4.7		8.5	17	1.42							
1091228	0929	142	4.0			13	1.32							

DATE	TIME	GLUCOSE	PAB	TRF	ZN	CU	KETONE	CK	CKMB	PT-P	PT-C	APTT-P	APTT-C	TRO-T
1091223	1452	95												
1091223	1454									10.2	10.3	29.0		

特殊檢查

病理報告

其它

出院時情況

- 3.改門診治療

出院指示

-

藥名	劑量	劑量單位	用法	頻次	數量	數量單位	現況	天數	單位
Olmesartan tab 20mg	1	TAB	PO	QD	14	TAB	TH	14	天
Ultracet tab	1	TAB	PO	BIDPRN	28	TAB	TH	14	天

出院計劃

主治醫師 2062F 蔣鋒帆 / 住院醫師 2331F 陳凱祥

正式摘要(電子簽章：000000206180392)

W52 / W52

doctorRank:B
